# Additive Manufacturing of Polymer Materials: Progress, Promise and Challenges

**DOI:** 10.3390/polym13050753

**Published:** 2021-02-28

**Authors:** Saad Saleh Alghamdi, Sabu John, Namita Roy Choudhury, Naba K. Dutta

**Affiliations:** 1 School of Engineering, Chemical and Environmental Engineering, RMIT University, Melbourne 3000, Australia; s3459013@student.rmit.edu.au (S.S.A.); namita.choudhury@rmit.edu.au (N.R.C.); 2 School of Engineering, Manufacturing, Materials and Mechatronics, RMIT University, Bundoora 3083, Australia; sabu.john@rmit.edu.au

**Keywords:** additive manufacturing, polymer, 3D printing, 4D printing, composite materials, smart materials, polymer composites

## Abstract

The use of additive manufacturing (AM) has moved well beyond prototyping and has been established as a highly versatile manufacturing method with demonstrated potential to completely transform traditional manufacturing in the future. In this paper, a comprehensive review and critical analyses of the recent advances and achievements in the field of different AM processes for polymers, their composites and nanocomposites, elastomers and multi materials, shape memory polymers and thermo-responsive materials are presented. Moreover, their applications in different fields such as bio-medical, electronics, textiles, and aerospace industries are also discussed. We conclude the article with an account of further research needs and future perspectives of AM process with polymeric materials.

## 1. Introduction

**Figure 1:**
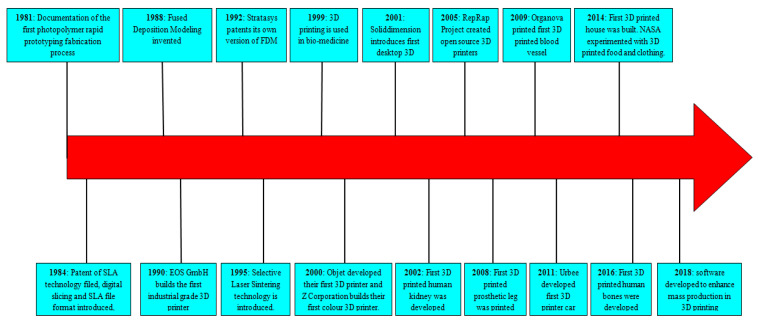
History and landmark achievements in 3D printing timeline from the 1980s to today.

**Figure 2:**
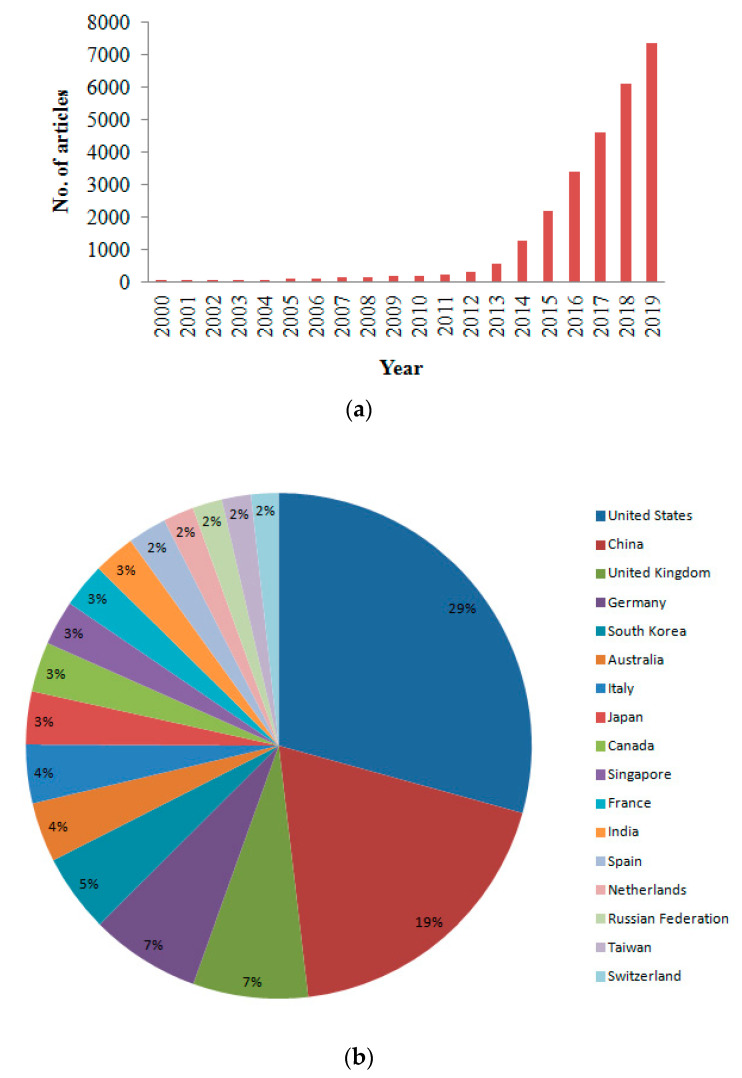
(**a**) Global scientific trends in additive manufacturing: a summary of the year-wise publications of research articles that are indexed in Scopus database. (**b**) Geographical distribution of patents on additive manufacturing as per Scopus database**.**

Additive manufacturing (AM), interchangeably termed as three-dimensional printing (3DP) is an emerging disruptive technology; which is currently stimulating innovations in design and engineering, materials and manufacturing, reducing cost and waste, and increasing efficiency. It is poised to reshape manufacturing; and has the potential to make marked industrial, economic, and societal impacts. Historically, the AM technology was introduced in the 1980s (Figure 1) and was initially limited only to small products’ manufacturing or prototyping. Since 2009, the development in AM technology has been very rapid (Figure 2) and has charted out new dimensions in engineering applications in diverse industrial sectors. Figure 1 shows the timeline of the key discoveries and the milestones in AM space from the 1980s to today. However, it is still at a young stage and evolving to develop quality complex components out of a variety of materials and even multimaterials with high levels of precision and performance.

AM essentially aims at building a three-dimensional product from a model generated by a computer aided design (CAD) and offers an unprecedented opportunity for digitization of the manufacturing sector. Using various software and technological platforms, enhanced computational power and connectivity, the scope of achieving enhanced flexibilities using this technology has increased dramatically. Moreover, it also aids in the improvement of design accuracy, reliability, and making inroads for customized manufacturing of products with near-infinite design flexibilities. It also has moved into printing large parts and large-scale production (https://www.3dnatives.com/en/essentium-190320195/ (accessed on 8 December 2020)). Its widest applications are in the automobile, aerospace, consumer goods, electronic, and biomedical fields. The introduction of fiber-reinforcement in the 3D printed plastics/resin products, to improve their mechanical performances, has augmented AM’s expansion in polymer composite manufacturing. This technology is burgeoning exponentially throughout the globe and is continuously used for novel applications, efficient production, innovative 3D printing materials, and at a cost-competitive price.

The bibliometric analysis from the Scopus database shows 30,700 bibliographic articles published in total in the last decades (2000 to 2019) (Figure 2a) on additive manufacturing/three-dimensional printing (AM/3DP). Moreover, in the same period globally 157,132 patents were filed on 3DP/AM. Global trends for the countries reveal a prominence of the USA and China in scientific literature and patent production in AM. The growth in publication on AM research is presented in Figure 2a. It is also observed from Figure 2b that the USA was the most prolific country, regarding 3D printing with 8705 publications, followed by China with 5603 publications. The detailed report on the country-wise publications on AM is presented in Figure 2b. Similarly, the maximum numbers of patents are filed in the United States Patent and Trademarks Office, with 86,708 patents, followed by the Japan Patent Office with 40,519 patents. Improvement of the performance of medical components, including tailored prostheses by providing unique and innovative designs using various advanced materials and hydrogels to improve the product quality and biocompatibility, is one of the major focus areas of AM technology. More recently, 4D printing has also emerged. It offers the capability to form complex 3D structures that could adopt different pre-determined forms and shapes when subjected to different environmental stimuli. The unique shape change mechanism, which is exhibited in this process is a combined effect of shape programming and the application of programmable smart materials such as stimuli-responsive polymers. AM is an evolving key enabling group of technologies that has the potential to create disruptive solutions to manufactured products directly from digital models without human intervention and complicated frameworks, and future possibilities are seemingly unbounded.

This review intends to be a brief account of the advances and the state-of-the-art AM manufacturing processes using polymer materials, which includes polymer composites, polymer nanocomposites, thermoplastic polymers, polymer hybrids, fiber-reinforced plastics, and biopolymers. For completeness, in some sections, we may include AM using ceramics, metals and multi-metals as appropriate. The advancement in the use of 4D printing using stimuli-responsive polymer materials that have resulted in great strides toward the generation of functional biomaterials for medical purposes has also been encapsulated. The review starts with a brief historical background and advances in the 3D printing process and equipment. Then, an attempt is being made to provide a succinct review of polymer-based 3D printing techniques, which include the methods used, materials employed, processes developed, followed by their applications in different industries. Finally, research gaps and challenges faced in using this technology and the prospects are presented. However, the 3DP space is very broad and this review may not capture all the details.

**Figure 3:**
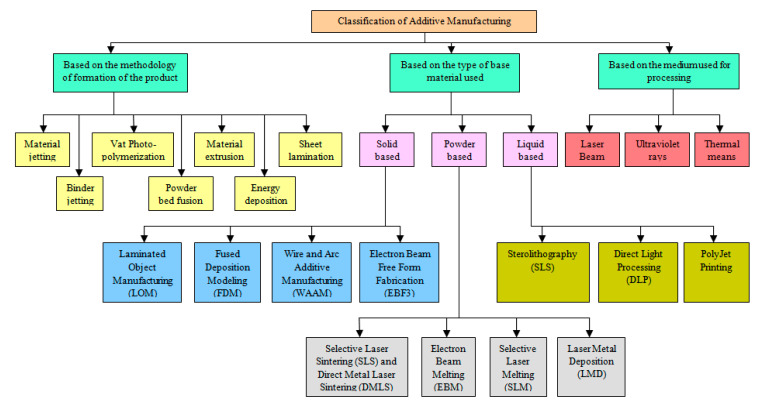
Classification of additive manufacturing processes from different contexts.

The AM process is fundamentally different from the traditional subtractive manufacturing process, and the structure is built into its designed shape using a ‘layer-by-layer’ approach. The process can be made suitable for most industrial sectors, using a wide range of materials; viz. metallic, ceramic, polymer materials along with combinations in the form of composites, hybrid and graded functional materials and hydrogels, in different physical states including solid, liquid, viscoelastic, and gels. Therefore, the AM process is very broad, and it may be classified in many different contexts. Figure 3 illustrates the broad classification of AM processes from different perspective. According to the American Society for Testing and Materials (ASTM F2792-12a), there are more than 50 different additive manufacturing technologies based on the above-classified processes [1]. Hence, ASTM has formulated a set of standards that classify the range of additive manufacturing processes into 7 general categories (ISO/ASTM 52900:2015). According to ASTM, based on the methodology of formation of the final components, AM processes can be classified into the following seven types, namely: (1) jetting, (2) binder jetting, (3) vat photopolymerization, (4) powder bed fusion, (5) material extrusion, (6) energy deposition and (7) sheet lamination [1,2] (Figure 3). AM may be further classified based on the physical state of the base material used and processed to form the product. This classification includes solid, liquid, and powder-based processes (Figure 3). It is also classified based on the medium used to process the base material, such as laser beam, ultraviolet rays, thermal means, etc. The AM processes are evaluated using many parameters such as fabrication speed, part strength, resolution, built volume, cost, quality, and surface finish. They are continuously evolving to make bigger and more complex products in the more flexible and economic way [2,3,4,5,6,7,8,9,10]. However, among these, the most critical parameters are the fabrication speed and the resolution. Therefore, the AM processes, which are classified based on the methodology of product formation are often evaluated based on these two process parameters. On this basis, a variety of 3D printing systems were developed and were successfully employed to produce several advanced, sophisticated structures for research and industry applications. These are briefly discussed in the following sections with the indication of the advantages and disadvantages of a specific AM process.

## 2. Classification of AM Processes

### 2.1. Material Jetting

Material jetting (MJ) is one of the fastest and most accurate 3D printing processes in which the liquid material droplets of the build and support materials are selectively jetted onto the build platform. These droplets soften the previously deposited material layer partially; thereafter it solidifies/cured with UV light as a single piece and removed from the platform. The MJ process is essentially analogous to a two-dimensional inkjet printer [11,12]. The materials used in this process are generally thermoset photopolymers (e.g., acrylics) that are available in a liquid form. A wide range of materials including acrylonitrile butadiene styrene (ABS)-like, rubber-like, and fully transparent materials are commercially available. Multi-material printing is a key strength of this process and is ideal for creating realistic visual and haptic prototypes with very smooth surfaces that resemble injection molded parts with homogeneous mechanical and thermal properties. However, MJ also has some limitations including: (i) poor mechanical properties, (ii) photosensitivity, and degradation of mechanical properties over time, (iii) high material cost that makes it financially not viable for some applications. Material jetted parts are mainly suitable for non-functional prototypes [13,14].

#### PolyJet Printing

**Figure 4:**
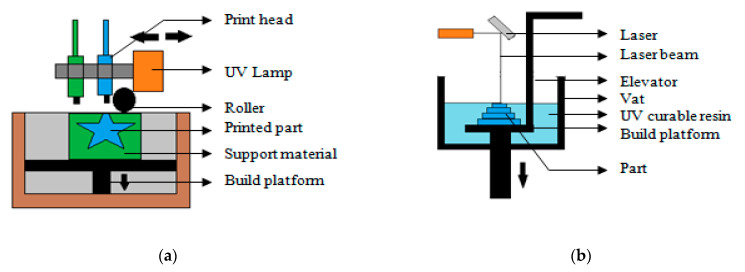

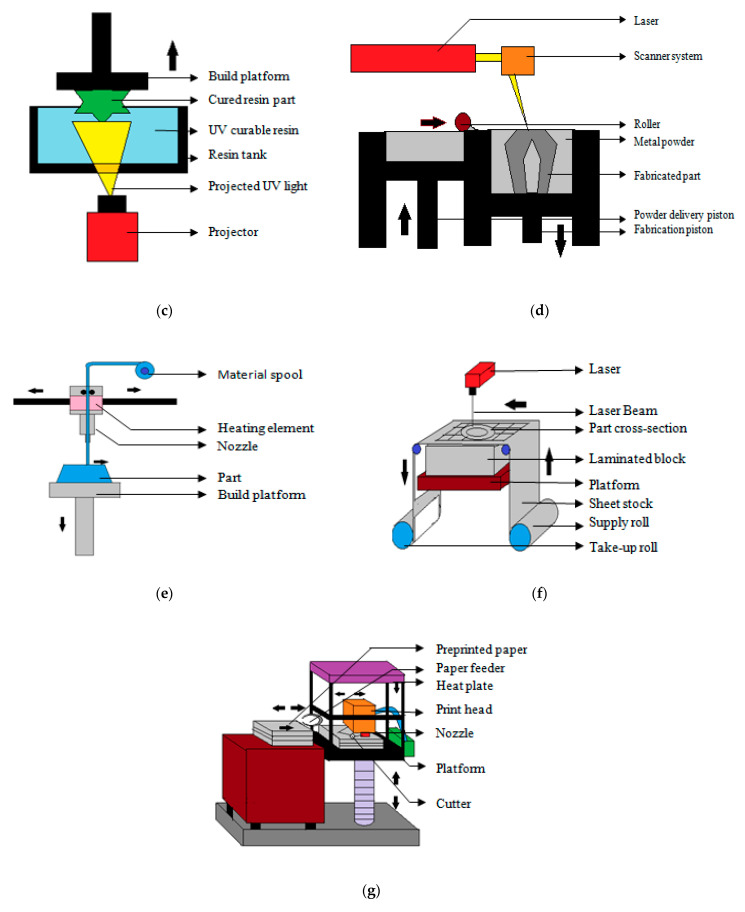
Schematic representation of the most popular additive manufacturing processes used for fabrication of polymer products (**a**) polyjet printing; (**b**) stereolithography (SLA); (**c**) direct light processing (DLP); (**d**) selective laser sintering (SLS); (**e**) fused deposition modeling (FDM); (**f**) laminated object manufacturing (LOM); (**g**) selective deposition modeling (SDM).

The polyjet printing process is essentially an inkjet technology that is used to create 3D parts. An inkjet head consisting of the photo-resin moves along X and Y axes and deposits the photo-resin according to the CAD file (Figure 4a). Thereafter, each layer is cured using an ultraviolet lamp until the part is completed [15]. Vdovin et al. [16] fabricated samples of acoustic metamaterials with high sound absorption. The samples in the form of cellular thin-walled periodic structures with links between the cells were synthesized by photopolymer material using the additive technology. The results of the experimental study of the synthesized samples demonstrated high sound absorption efficiency. Kitamori et al. [17] developed a biocompatible class VI resin polyjet photopolymer mouthpiece using polyjet printing, which was used as a fixation device for head and neck radiotherapy patients. Hong et al. [18] developed a computer tomography (CT) image-based 3D-printed model of thyroid cancer using various kinds of 3D printers including polyjet printers and compared their accuracies and other aspects regarding facilitating this patient-physician communication.

### 2.2. Binder Jetting

Binder jetting (BJ) is an AM method in which the layers of powdered particles selectively deposited onto the build platform are joined by depositing a liquid bonding agent using CAD to form the part. In BJ, the printer head is designed for dropping the bonding liquid on the powdered particles that are placed on the platform. When one layer is formed, the platform is moved down to form the next layer. The primary advantages of this technique include: (i) accommodating complex design, (ii) freedom from support structures, and (iii) higher printing speed. It can incorporate a wide variety of materials including polymers, metals, sands, and ceramics of different colors. Moreover, it is capable of building large, complex components at relatively low-cost than many direct 3-D printing processes; and is capable of manufacturing high-value products with structurally robust materials. BJ technology has matured significantly in recent years; however, further research is essential to generate the fundamental data required for the robust implementation of this technology on an industrial scale [19,20,21,22,23].

### 2.3. Vat Photo-Polymerization (VPP)

In this method, a vat of liquid photo-curable resins is used, out of which solid products are constructed using a suitable laser to selectively harden the photosensitive liquid into 3D solid in a layer by layer format. In the process, the cured part either descends into or ascends from the vat of photosensitive liquid resin. This is a common rapid manufacturing and rapid prototyping technology for producing parts with incredibly high resolution and excellent surface finish but can be more expensive and prone to increasing brittleness over time. It can be used to create concept models, rapid prototypes, and complex parts with intricate geometries. The *Z*-axis layer height, which is commonly used to define the resolution of a 3D printer can be adjusted in between 25 microns and 300 microns on modern VPP printers (e.g., Formlabs SLA (stereolithography) 3D printers), with a trade-off between speed and quality [24,25,26]. The development of highly sophisticated compact SLA 3D printers along with innovative SLA resin formulations with a wide range of optical, mechanical, and thermal properties to match those of standard, engineering, and industrial thermoplastics offer opportunities to accelerates innovation and supports businesses across a wide range of industries, including engineering, manufacturing, dentistry, healthcare, education, entertainment, jewelry, and audiology [27].

#### 2.3.1. Stereolithography

Stereolithography (SLA) is the first developed AM process. In this process, the part is formed by exposing a photosensitive polymer resin to an ultraviolet laser (Figure 4b). When the photosensitive resin comes in contact with an ultraviolet laser, it solidifies to form the layer. Subsequently, layers are formed by exposing the resin as per the CAD data while the build platform is lowered. Micro-stereolithography is another version of this process with higher resolution [28]. SLA may be used to fabricate product using diverse range of materials. For example, Liu et al. [29] demonstrated ZrO_2_–Al_2_O_3_ composite ceramic parts using UV curable ZrO_2_–Al_2_O_3_ composite ceramic pastes based on the SLA process with subsequent debinding and sintering process. Karakurt et al. [30] created personalized drugs and medicines geometries with novel biocompatible photochemistry consisting of ascorbic acid (AA) encapsulated in a poly(ethylene glycol) dimethacrylate (PEGDMA)-based polymer network and polymerized using riboflavin as a photoinitiator. In this work, they have shown that by using novel biocompatible photochemistry and 3D printing it is possible to successfully load and release ascorbic acid as a model agent and thereby opening up a new class of manufacturing protocols to encapsulate ascorbic acid and other water-soluble vitamins for drug delivery applications. Xu et al. [31] used this process to manufacture a multi-layer 3D printed oral dosage form (polyprintlet) incorporating four antihypertensive drugs including irbesartan, atenolol, hydrochlorothiazide, and amlodipine. Gallup et al. [32] fabricated nasopharyngeal swabs using this process [32]. The use of passive stabilization to support SLA printing aboard a moving vessel at sea has been demonstrated by Philips et al., which laid the foundation for lithographic 3D printing in seagoing oceanographic and naval applications [33]. Zhou et al. [34] developed β-TCP bio-ceramics for the hard tissue repair applications using SLA. The fabricated samples exhibited good fluidity, uniform dispersion, and good stability.

#### 2.3.2. Direct Light Processing

Direct light processing (DLP) is a similar process as stereolithography. However, the only difference is that, in this process, a DLP projector is the source for curing the photo-resin instead of ultraviolet light (Figure 4c) [35]. Beside polymers, DLP has been employed for a variety of biomedical applications using ceramics, hydrogels and bioinks. Komissarenko et al. [36] explored the printability of scandia-stabilized zirconia ceramic parts using a DLP 3D printer. They demonstrated that the DLP is a promising method of fabricating scandia-stabilized zirconia parts. Shen et al. [37] developed a photocurable chitosan bioink (CHI-MA) to be used for DLP technology. Through the DLP printing, CHI-MA bioink was processed into complex 3D hydrogel structures with high resolution, high-fidelity and good biocompatibility. Bagheri et al. fabricated scaffolds for bone grafting purposes using this technology with bio-composite materials [38]. Recently, all-ceramic teeth have been fabricated from ZrO_2_ for biological engineering using photosensitive resin-based this technology [39]. Hard tissue scaffolds with poly L-lactic acid (PLLA) resin compatible with this process has also been used [40]. The results confirmed that the synthesized polymer and the DLP method of 3D printing were suitable for fabricating scaffolds with intricate structures. Mao et al. [41] developed liver-specific bioinks by combining photocurable methacrylate gelatin (GelMA) with liver decellularized extracellular matrix (dECM), and human-induced hepatocytes (hiHep cells), which were encapsulated to form cell-laden bioinks. The mechanical properties of the GelMA/dECM bioinks were carefully tuned before 3D printing. Subsequently, the DLP-based bioprinting was used to fabricate the liver microtissue to help restore hepatic functions. Kardy et al. [42] assessed the feasibility of using DLP 3D printers in fabricating solid oral dosage forms. The study demonstrated that DLP 3D printers can be used as a platform for fabricating oral tablets with well-defined shapes and different release profiles. Xu et al. [43] fabricated bioactive glass-ceramics (AP40mod) as a scaffold containing endothelial progenitor cells (EPCs) and mesenchymal stem cells (BMSCs) to repair critical-sized bone defects in rabbit mandibles using DLP technology. A new flexible approach to read out light-addressable potentiometric sensors (LAPS) has been developed by Wagner et al. with the help of this process [44]. The DLP technique has also been used to fabricate polymer-based microfluidic chips [45]. Hong et al. [46] demonstrated that silk fibroin as a natural polymer fabricated with glycidyl-methacrylate (Silk-GMA) for DLP 3D printing. The ability of chondrogenesis with chondrocyte-laden Silk-GMA was evaluated in-vitro culture system and applied in vivo. The DLP 3DP system provided a 3D product with even cell distribution due to the rapid printing speed and photopolymerization of the polymer. To develop microporous channels, a photosensitive epoxy-acrylic siloxane system was employed as the matrix for the DLP and a nonphotosensitive liquid siloxane was introduced as an additive to ensure channel formation during pyrolysis in a post processing step [47]. The results showed that the DLP 3D printed SiOC lattices with the addition of liquid siloxane show a better macro-appearance and macro-mechanical property with scarifying its micro-hardness.

### 2.4. Powder Bed Fusion (PBF)

Power bed fusion (PBF) is an AM process where thermal sources like laser beams are used to fuse the powdered particles in a predictable way to design objects using CAD. The formed layer is made uniform using a recoater blade and thereafter the next layer is formed. There are several variants of the PBF process, which are designated by the heat source used and the type of material employed. The most dominant categories of this process are: (i) selective laser melting (SLM), (ii) selective laser sintering (SLS) and (iii) electron beam melting (EBM). Each variant offers advantages and disadvantages, so suitability is weighed on the application basis [48,49]. SLS is a trademarked term, and is also known as laser beam PBF (PBF-LB). In the SLS process, powdered polymer materials such as nylon or polyetherketoneketone (PEKK) are sintered and complex 3D parts are generated by building successive layers of powder materials over each layer the powder particles are sintered using a laser beam in order to form a layer (Figure 4d). The layers are built continuously until the final part is completely built. SLS with higher resolution in micro-scale is known as micro selective laser sintering (µ-SLS). This process can produce feature size resolution of less than 5 µm [50]. Its main applications include microscale fabrication of sensors, actuators, micro-optoelectronic components, etc. In SLM, a laser is used to provide heat and fully melt the powder rather than sintering it as observed in SLS. The process is generally applied to metal powders such as aluminum alloys; titanium and its alloys; and stainless steel. In the process, an inert atmosphere (typically argon) is included in the build chamber to prevent oxidation and/or nitriding of the consolidated material. EBM is a comparable process to SLM, where the laser is replaced with an electron gun (hence a PBF-EB process). In the EBM process the build chamber utilizes a vacuum instead of an inert atmosphere. The other two less popular PBF techniques are respectively (iv) Fused with agent and energy and (v) thermal powder bed fusion. HP’s multi jet fusion (MJF) comes under the fourth category, where the powder bed is heated uniformly at the start, where a fusing agent is used to bond the powder to create 3D geometrical parts. Danish company Blueprinter uses thermal fusion for selective heat sintering (SHS) for sintering thermoplastic powder to create 3D parts [51,52,53,54]. The PBF process has several advantages including, (i) reduced material wastage, (ii) reduced product development times, (iii) rapid prototyping and improved low volume production, (iv) building functionally graded parts, (v) fully customized parts and eliminating fixed designs, (vi) good resolution, (vii) efficient recycling of unused powder, (vii) design with multilateral-ability to join many material grades, including ceramics, glass, plastics, metals and alloys, (viii) elimination of the need for machining fixtures and (ix) increase productivity through filling the build area with multiple parts [49]. Some of the major disadvantages are: (i) relatively slow and long print time, (ii) often post-processing/post removal is required steps are generally required for optimization of the final properties, e.g., improve the mechanical property, reduce residual stress, and improved surface finish, (iii) weak structural properties/surface texture, as the parts are made fusing metal powder, (iv) surface quality is dependent on the grain size of the powder, (v) possibility of thermal distortion, (vi) increased cost related to high power usage [15].

### 2.5. Material Extrusion

Material extrusion (ME) is an AM process in which a spool of material (usually thermoplastic polymer) is pushed through a heated nozzle at a constant pressure in a continuous stream and the requested materials are selectively deposited, layer by layer, as per the design to fabricate the 3D parts (Figure 4e). It is also known as fused filament fabrication (FFF) and is one of the most popular processes for AM. The technology was commercialized in 1990 by the Stratasys company (https://www.stratasys.com (accessed on 8 December 2020)) and the proprietary term fused deposition modeling (FDM) was adopted. A wide variety of materials can be extruded using FFF; the most commonly used materials being thermoplastics such as acrylonitrile styrene acrylate (ASA), acrylonitrile butadiene styrene (ABS), polycarbonate, polyetherimide, polylactic acid (PLA) high-impact polystyrene (HIPS), thermoplastic polyurethane (TPU), aliphatic polyamides (PA, Nylon), and high-performance plastics such as polyether ether ketone (PEEK), and polyetherimide (PEI) [2,55,56,57]. Due to the simplicity, reliability, and affordability of the FDM process, it has been widely adopted by different industries, academia, and consumers for the fabrication of both prototypes and functional components using both commodity and engineering plastics [58,59,60,61]. This process requires preformed fibers that have a uniform size and material properties for feed through the rollers and nozzle. Once the filament is extruded out, it solidifies due to loss of heat and fuses with the layer beneath it. When one layer is built in the cycle of printing, the build plate is either lowered down or the print head is lifted in order to print the next layer. This technique (Figure 4e) enables the creation of complex-shaped parts by extruding the material through nozzle/s, which may be fitted with one or more nozzle heads. In most of the FDM systems, two nozzles are present. One of the nozzles is used for modeling the part and the other is used for modeling the support material [61]. The support materials can be removed easily by post-processing such as water jetting once the printing is completed. There are many varieties of FDM machines. In some FDM machines, the print-head travels within the XY plane, and the bed travel in the Z direction (Cartesian FDM). In another type, the printer-heads move in XYZ directions, and the platform bed is stationary (polar FDM). In yet another type, the print-heads travel in the printing environment (delta FDM). These printers may differ in properties such as print speed, resolution, and print quality [58].

Besides traditional plastic components, this process has been adopted for a wide range of industries for innovative product development including soft magnets [62], drug delivery systems in the pharmaceutical field including personalized medicines [63,64,65,66], in the forensic comparative analysis [67], in complete electrochemical sensing devices fabrication [68], in 3D printed microfluidics [69] and to design and manufacturing of complex porous scaffolds for biomedical, and tissue engineering applications [70]. Moreover, it also fits well in the framework of primary and secondary recycling. The major advantages of FDM include: (i) creation of concept models/functional prototypes at reduced costs and shorten development timelines; (ii) fabrication of end-use parts without the expense and lead time of traditional tooling or machining, (iii) fabrication of manufacturing tools at reduced cost without expensive machining or tooling, (iv) flexibility to the material in processing and handling; (v) market product faster; (vi) no unbound loose powder and (vii) there is no solvent removal requirement.

Additionally, paste-like materials such as ceramics, concrete, and chocolate can also be extruded using this 3D printing technique. Recently, progress on the ink material extrusion, known as liquid deposition modeling (LDM) have also been advanced [71]. The term composite filament fabrication (CFF), coined by Markforged (https://markforged.com/3d-printers (accessed on 8 December 2020)) for composite fabrication is also essentially an FDM process, where a 3D printer is equipped with multiple extruders for opening multi-material capabilities to the 3D print composite material and/or to speeding up the fabrication process. In a typical CFF with two nozzles, one nozzle operates following the typical material extrusion process- lays down a plastic filament that forms the outer shell and the internal matrix of the part, and the second nozzle deposits a continuous strand of composite fiber (e.g., carbon, fiberglass, or Kevlar) on every layer. These continuous strands of composite fibers inside 3D printed parts add strength to the built object that is comparable to parts made of metal with precision design and construction yields predictable, repeatable results [72].

### 2.6. Directed Energy Deposition

Directed energy deposition (DED) is a relatively complex 3D printing process and share similarity to PBF technologies; it uses a focused energy source, such as a laser or electron beam to melt the material before selectively depositing, layer by layer, as per CAD. The material is melted at the same time as it is deposited by a nozzle and the technology is at the frontier of material extrusion and powder bed fusion. The material used in this process include powders of metals such as copper, stainless steel, titanium, tin, aluminum, nickel, and cobalt [2,73,74]. This technology is often referred to by other names such as laser engineered net shaping (LENS), direct metal deposition (DMD) and electron beam additive manufacturing (EBAM), depending on the specific application or method [75]. The material fed to the nozzle is either in powder or wire form and the process is typically used with metals and less for polymers. For metals, almost any metal including titanium and titanium alloys, tantalum, tungsten, niobium, stainless steel, aluminum, that is weldable can be 3D printed with DED. The wire used typically ranges from 1–3 mm in diameter and powder particle sizes are similar to those used in powder metallurgy processes, between 50 and 150 microns. It may also be used with polymers and ceramics. For example, AREVO (https://arevo.com/products?lang=en (accessed on 8 December 2020)) uses polymer DED with a filament of carbon fiber to fabricate strong, lightweight reinforced composite parts. In this process, the thermoplastic filament is melted by a heat source and compacted by a roller to generate the layers of the object. It allows to produce relatively large parts with minimal tooling. Furthermore, it also allows the use of multiple materials with different compositions to fabricate products with composition gradients or hybrid structures. Typically, DED is significantly faster and less expensive than PBF. The advantages of DED are evident in that material use as well as cooling and build times are greatly reduced compared to PBF. Moreover, DED is increasingly replacing conventional methods for the repair of parts particularly for complex and precise parts, e.g., for applications such as the repair of damaged turbine blades or propellers [76].

### 2.7. Sheet Lamination

Sheet lamination is an AM process, where typically, 3D objects are formed by stacking and laminating thin sheets of material. The lamination method can be (i) bonding, (ii) ultrasonic welding, or (iii) brazing, and the final shape is achieved either by laser cutting or CNC machining. The final product can be made either using: (i) the form then bond process—first the sheet material is cut to shape and then bonded to the previous layer to create a 3D geometry—or (ii) the bond then form process—the sheet material layers are bonded together before cutting them into the desired shape. The materials used may include paper, plastic, and metal sheets. Different types of sheet lamination techniques are used for different purposes and they are directly tied to the individual process. The process can also be categorized based on the lamination technique used to bond the sheets together such as (i) laminated object manufacturing (LOM), (ii) ultrasonic additive manufacturing (UAM), or (iii) plastic sheet lamination (PSL). The UAM process uses sheets or ribbons of metal, which are bound together using ultrasonic welding.

LOM is one of the first commercialized (1991) additive manufacturing techniques, use a similar layer by layer approach but uses paper as material and adhesive instead of welding and is discussed in detail in the following section. LOM is an additive manufacturing process, which is carried out by cutting and lamination of sheets or rolls of materials such as paper, polymer composites, ceramics, and tapes filled with metals (Figure 4f). Recently, different modification of LOMs has been developed further for manufacturing complex phase materials. Luong et al. [77] developed a novel 3D laser-induced graphene (LIG) foam printing process based on LOM, which was combined with the subtractive laser-milling process to yield further refinements. The LIG graphene foams developed using the process showed good electrical conductivity and mechanical strength and demonstrated potential for energy storage and flexible electronic sensor applications. The products fabricated using the LOM process have the potential to result in thermal residual stresses and deformations due to materials mismatch among layers and gradient cooling [78]. The feasibility of preparing silicon nitride ceramics components by aqueous tape casting in combination with LOM has been demonstrated by Liu et al. [79]. Such samples showed good flexural strength. Krinitcyn et al., [80] synthesized MAX-phase (M_n+1_AX_n_—where M is an early transition metal, n = 1, 2, or 3, A is an A-group element (specifically, the subset of elements 13–16), and X is carbon and/or nitrogen—that bridge the gap between the metals and ceramics) components using the LOM process. Zhang et al. [81] developed a frozen-slurry-based LOM-slurry, composed of water, alumina powder, and organic binder, that was used to fabricate porous ceramic structures. The water in the slurry crystallized to obtain good support strength, which was further cut to obtain the required 2D pattern using a laser. Thereafter, they were stacked layer by layer and freeze-dried to obtain the porous structure.

Selective deposition modeling (SDM) is another additive manufacturing process, in which each layer is bonded with the previous layer using a selective application of adhesives according to the CAD data (Figure 4g). Further, the selective layer is cut as per CAD data and the process is repeated until the entire part is completed. This method is capable of producing full color 3D printed parts. SDM is an ideal choice for concept models, that enables to produce highly stable tactile models and prototypes. Hung et al. prepared highly order flexible layers of graphene oxide on modified polyacrylonitrile substrates using this process [82]

PSL uses heat and pressure without the adhesive as it relies on melting the sheets together. Laminated objects are often used for aesthetic and visual models and are not suitable for structural use. UAM uses metals and includes aluminum, copper, stainless steel, and titanium. Selective lamination composite object manufacturing (SLCOM) is employed to fabricate composite objects using carbon fiber, fiberglass, aramid fiber along with metal fibers like steel, aluminum, or titanium. The process is low temperature, requires relatively little energy, can bond different materials, and is relatively low cost as it uses standard material. However it has many disadvantages including: (i) part resolution is linked to sheet thickness (ii) requires post-processing to achieve desired product, (iii) limited material options available, (iv) generates a lot of waste, (v) bonding strength depends on the laminating technique used [83,84,85,86,87]

## 3. Additive Manufacturing Using Polymers

AM using polymers can be carried out successfully using a variety of polymer materials in different forms and compositions including their composites, nanocomposites, continuous/discontinuous fiber-reinforced thermoplastic composites, and hybrids. The most widely used polymer 3D printing methods and processing techniques used across polymer industries along with major global 3D-printer manufacturer are highlighted in Table 1.

The most common thermoplastic polymer materials used in FDM processes are acrylonitrile butadiene styrene (ABS), polylactide (PLA), polyamide (Nylon), nylon 12, polycarbonate (PC), glass-filled nylon, and epoxy resin. Some of the commercial polymer materials that are most popular in the FDM process include ABS_M30 and polycarbonate (PC) [88]. In SLS and multi-jet, most commonly Polyamides (Nylon PA 11 and Nylon PA 120 and thermoplastic polyurethane (TPU) are used [89]. For the SLA technique various photo-active special polymers have been developed. For example, Somos^®^ ProtoGens, a liquid, ABS-like, photopolymers (commercial product from DSM, www.dsm.com/somos (accessed on 8 December 2020)) are the first SLA resins to demonstrate different material properties based on machine exposure control. Based on oxetane chemistry, this high-temperature resistant, ABS-like photopolymer offer opportunity to build three-dimensional accurate parts ideal for general purpose applications. They provide considerable processing latitude and are ideal for the medical, electronic, aerospace, and automotive markets that demand accurate durable concept models, highly accurate, humidity and temperature resistant parts. SOMOS water clear ultra 10122A is another commercially available optically clear stereolithography material (from DSM) tailored for colorless, functional parts with excellent temperature resistance. In the polyjet technique parts are built using liquid photopolymer droplets, that are cured with UV light. There are numerous polyjet materials including biocompatible plastics, which can be used in medical technology are commercially available. Furthermore, materials can be blended and mixed to obtain new properties. Durus White (simulated polypropylene material from Stratasys), Vero (rigid plastic with a smooth surface and available in a variety of colors and transparent, Stratasys), Tango (a soft, rubbery polyjet material), digital ABS (simulated ABS with comparable properties), RGD 525 are used in polyjet printing process. Typical properties of AM components manufactured using various commercial polymers based on the data sheets of EOS GmbH; Stratasys, USA, and SYS are presented in Table 2.

The polymers used in AM processes can be rationally classified as thermoplastics, particle reinforced polymer composites, polymer matrix composites, fiber-reinforced polymer composites, thermo-responsive polymers, nanocomposites, and thermoplastic elastomers composites. Recent developments in AM in different major areas of the polymers are discussed below.

### 3.1. 3D Printing of Thermoplastics

Thermoplastics are melt-processable plastics that have the ability to become softened on heating and solidify on cooling, repeatedly; and during the process, they also retain their inherent properties [90]. Traditionally, processes such as material extrusion and injection molding are used for producing thermoplastic parts. However, the development of 3D printing technologies such as FDM and SLS has made it possible to create complex thermoplastic parts using AM. In these methods, the thermoplastics are heated to reach a malleable state and then extruded onto a platform, where the solidification takes place [91]. Thermoplastics are mainly used due to their rigidity, high strength, and high-temperature tolerance and stability. Some of the common thermoplastics used are acrylic, ABS, nylon, PLA, PC, PE, etc. as shown in Table 1 [92].

AM processes such as SLA, SLS, and FDM are used to develop various components, which can be used in various general purpose and engineering applications, and specialty biomedical applications. Gkartzou et al. [93] have demonstrated 3D printing processes for processing bio-based blends of polylactic acid (PLA) using FDM. Furthermore, the parameters, which contribute to the stress imposed on melt and shear rate such as printing speed, extrusion temperature, and width of the fiber were examined. Childs et al. reported thermal and powder densification modeling of amorphous polycarbonate using SLS [94]. Lee et al. developed polypropylene fumarate (PPF) with various porosity to study its suitability as scaffolds in tissue engineering [95]. Matellan et al., have developed polymethylmethacrylate (PMMA) microfluidic devices, which are used in biomedical applications [96]. 3D printed water-soluble scaffolds have also been developed using PDMS microfluidic chambers [97]. Some craniofacial and dental scaffolds are also demonstrated using a polymer system [98]. Valtonen et al. replicated nasal cavities in order to study the air passage through them for clinical applications [99]. The biomedical applications were demonstrated for easy fabrication of complex biomedical structures in a cost-effective way. Apart from biomedical applications, polymer-based additive manufacturing processes are also demonstrated in complex prototype formations. Moreover, the polymers are also combined with reinforcements in order to improve their mechanical properties.

### 3.2. 3D Printing of Polymer Matrix Composites

Polymers are generally preferred for AM processes due to their adaptability to different processes and it can be customized to complex geometries with high accuracy [100]. However, the polymers have inferior mechanical properties and are not suitable for many load-bearing applications. Therefore, research have been undertaken to overcome the disadvantages of polymers. Polymer matrix composites (PMCs) are generally comprised of various fibers, which are either short or continuous, that are bound together by using an organic polymer matrix. The reinforcement of polymers with fibers and particles was introduced to increase the mechanical properties of the polymer so that they can be used in load-carrying applications. These polymers include synthetic and biomaterial resins [101]. Polymers are reinforced using carbon-based materials. FDM has been commonly used for the fabrication of such reinforced polymer composites. Some of the reinforcement materials used in FDM are TiO_2_, carbon nanofibers, montmorillonite clay, graphene, etc. The reinforcements used in the SLA process are carbon nanotubes, graphene oxide, TiO_2_, BaTiO_3_, etc. Many polymer matrix composites have been studied for AM process by researchers. Sanchez et al. [102] studied the development and characteristics of acrylonitrile styrene acrylate (ASA) with carbon fiber. The authors observed that 20% in weight (wt %) of carbon fiber increased flexural Young’s modulus by 350% and thermal conductivity by 500%. Another commonly used reinforcement is glass fiber. It is used as its composition with polymer and the orientation of the fibers make it suitable for load carrying applications. The functional composites of glass fiber reinforced composites are equal to steel and it also has higher stiffness than aluminum [103]. Carneiro et al. [104] reported the characteristics and influence of orientation and layer thickness using glass-reinforced polymer composite using FDM. They observed that FDM is suitable for producing small parts and components.

One of the most important requirements of the reinforcements is desired interfacial adhesion. This property is required so that enhanced affinity can be achieved between fiber and resin matrix. It is also required to increase the durability of glass fiber reinforced polymers. In order to achieve this property, coupling agents are used to bond the fiber with polymers [105]. Fernandes et al. [106] studied composites made of short sisal fiber and polyethylene-graft-maleic anhydride. In this study, alkali treatment was used to improve the adhesion between fiber and matrix. Thereafter, the composite material was produced using a twin-screw extruder and compression molding. It was observed that 10 wt % of sisal fiber along with 2 wt % coupling agent shows improvement in flexural and tensile properties of the composite. Turk et al. [107] suggested four design principles of additive manufacturing that could improve the production of composites. The design principles include position and fixation elements, structural handling aids, layup and handling aids, and post-processing aids. Some of the polymer composites literature are shown in Table 3.

#### 3.2.1. Particle Reinforced Polymer Composites

Particle reinforced composites consist of the dispersion of particles of one material into the matrix of another material. These particles may vary in shape, size, and morphology. However, they are generally sphere, oval, polyhedron, or irregular in shape. The process of forming particle composite comprises of adding particles to the liquid matrix, which solidifies later, growing them in place using procedures such as age hardening, pressing together, and then inter-diffused by means of a powder process, etc. The composite powders are prepared by processes such as cryogenic ball milling, emulsion-precipitation, wet grinding-rounding, spray drying, and dissolution-precipitation [112]. The morphology, size distribution, constituents, and distribution of particles within the matrix is determined by the preparation process. The particle reinforced polymer composites are classified into dispersion strengthened and particulate reinforced composites based on the strengthening mechanism [113]. In dispersion strengthened composites, the particles are small in the size range of 0.01–0.1 µm. In this type, the strengthening occurs at the atomic or molecular level. Particulate composites are another class of particle reinforced composites in which coarse particles are dispersed in the matrix material. In such composites the interfacial interaction between the dispersed phase and the matrix phase is critical to determine the final properties.

Korhonen et al. [114] demonstrated the fabrication of graphene-based structures using stereolithography. The fabricated composite was further pyrolyzed in order to aid the reduction of graphene oxide to graphene in order to the electrical conductivity. The electrical conductivity achieved was in the range of the semiconductors. Rupp et al. [115] reported 3D printing of supramolecular polymers. 3D printing of supramolecular polymersand examined the impact of nanoparticles and phase separation on printability. The supramolecular polyisobutylene polymers showed rubber-like behavior. They fabricated nanocomposites of polyisobutylene by mixing 5–15 wt % of silica nanoparticles of size ~12 nm. The composite formed improved shape persistence and demonstrated high structural stability. Abedini et al. [116] formulated a hybrid model in order to enhance the strengthening behavior of particle reinforced composites. The hybrid model demonstrated that nonlinear behavior exhibited by the composite, when subjected to uniaxial tension, which depends on the size and volume fraction of the particles. Similarly, Yuan et al. [117] demonstrated the fabrication of flexible 3D soft metamaterials using thermoplastic polyurethane powders. The authors also demonstrated the fabrication of polymeric composites with multi-walled carbon nanotubes (CNTs) using SLS process. The composites developed were observed to have better heat conduction and heat absorption. It was also observed that the addition of CNTs enhanced the tensile strength, toughness, and elongation at the break without reducing the tensile modulus [118]. The general trend in final properties and 3D techniques used related to particle reinforced polymers is summarized in Table 4.

#### 3.2.2. Fiber Reinforced Polymer Composites

Fiber-reinforced composites (FRC) are composite building materials that consist of three components namely the fibers, the matrix, and the interface. Composite materials are produced by combining two or more materials with different properties so that better properties that cannot be achieved by either fiber or matrix separately are achieved. The mechanical properties of the fiber-reinforced composites are dependent on the strength and modulus of the fiber, chemical stability, and the quality of the interfacial bonding between the fiber and the matrix. Many additive manufacturing process technologies have been developed for the manufacturing of FRP composites that include: fused deposition modeling (FDM), laminated object manufacturing (LOM), stereolithography (SLA), additive gypsum printing manufacturing, ultrasonic additive manufacturing (UAM), fiber encapsulation additive manufacturing (FEAM), selective laser sintering (SLS) [119,120].

One of the most commonly used fiber used for reinforcement is carbon fibers. They provide low density, low coefficient of thermal expansion, and better thermal conductivity. The final material properties are significantly improved by using carbon fibre in AM process. It reduces the manufacturing time for producing functional parts when compared with conventional technologies. It also reduces warping and enables a larger build envelope [121]. Shi et al. [122] reported a dynamic capillary-driven additive manufacturing approach for manufacturing of continuous carbon fiber composites. This approach offers control over viscosity and degree of curing of carbon fiber composites. It enables infusion and curing of composites simultaneously in order to enable in situ solidification of the composites. Using this method, the printed composite was observed to have a high fiber fraction of 58.6% and a degree of curing of 95%. It also demonstrated high mechanical strength (~810 MPa) and modulus (108 GPa) of the product. In general, even though additively manufactured composites enable design flexibility and low cost, they suffer from low strength and stiffness when compared with conventionally manufactured composites. To overcome this limitation, Nawafleh et al. [123] developed a vibration integrated auger extrusion system and demonstrated it using carbon fibers. This method is a direct-write additive manufacturing technique, which allows the manufacturing of composites reinforced with short fiber. It aids in achieving better compression strength, flexural strength, and stiffness, and higher fiber volume ratio. Apart from glass fibers and carbon fibers, another commonly used fiber is Kevlar [124]. Dickson et al. [125] investigated the performance of composites reinforced with fiber using continuous fibers of carbon, glass, and Kevlar. The composites were fabricated using FDM technique. The influence of type and orientation of fiber and volume fraction were studied, and it was observed that maximum tensile strength was achieved using glass fibers. The key observation in final properties and 3D techniques used related to fiber reinforced polymer is illustrated in Table 5.

#### 3.2.3. Nanocomposites

The combination of nanotechnology and AM enables manufacturing of 3D parts with optimized properties and multi-functionality. The incorporation of nanomaterials such as carbon nanotubes improves the electrical conductivity, mechanical strength, and electromechanical and chemical sensitivity [126]. A variety of 3D nanocomposites are manufactured using AM technologies for various applications in different fields including microelectromechanical systems (MEMS), microfluidics, engineered materials and composites, micro-electronics, lab-on-a-chip, biosystems, and tissue engineering. The common additive manufacturing methods used for manufacturing nanocomposites include micro-stereolithography, extrusion-based technologies, inkjet printing, and powder bed technologies.

Bustillos et al. [127] developed polymer and boron nitride nanoplatelets composites using stereolithography. The nanocomposites were evaluated and revealed that addition of nanoplatelets increase microhardness, damping, and compressive strength. It was also found that the interaction of nanoparticles with the wavelength of the laser during the curing process is the most important factor in manufacturing 3D composites with improved functional properties. Postiglione et al., [128] developed nanocomposites, which were fabricated using UV-curable polymeric resin and various inorganic fillers via 3D printing. An investigation of the influence of filler concentration on the rheological properties was performed and the optimal printability parameters were identified. Abshirini et al. [129] developed polydimethylsilixane (PDMS) nanocomposite with electrically conductive properties for strain sensing. Multi-walled carbon nanotubes were uniformly distributed in PDMS and the developed nanocomposite was characterized using scanning electron microscope (SEM) in order to evaluate the microstructural features. Moreover, the strain sensing capability was evaluated by subjecting the specimen to cyclic tensile loading and was observed that the measured material strain had high fidelity. It was also tested in-situ to monitor the deformation of the human wrist joint. Zhang et al. [130] fabricated a photothermally responsive hydrogel nanocomposite of poly(N-isopropyl acrylamide) and graphene oxide using 3D printing. Wang et al. [131] demonstrated 3D printing of nanocrystal reinforced nanocomposites with methacrylic cellulose using stereolithography. It was observed that the developed composites exhibited better thermal stability and mechanical properties and good dispersion of particles. Chizari et al. [132] investigated the fabrication of nanocomposites using CNTs, which have high conductance with PLA. They fabricated scaffold structures, which could be used in liquid sensors. Yang et al., [133] demonstrated biomimetic anisotropic reinforcement architectures using electrically assisted nanocomposites 3D printing. In this work, they fabricated artificial meniscus using circumferentially aligned multi-wall CNTs. The printed meniscus showed enhanced mechanical properties and was found a promising replacement for the native meniscus tear problem. Invernizzi et al. [134] fabricated cationic radical free photopolymer using silica nanoparticles using stereolithography. It was observed that the nanoparticles provided a reinforcing and toughening effect to the photopolymer. The mechanical properties were also improved. The nanocomposites are widely used in the field of bio-structural, electronic, and electromagnetic fields [135].

### 3.3. Thermoresponsive Polymers

Responsive polymers have the capability to alter the chemical and physical properties when it is exposed to external stimuli including temperature, pressure, pH, ion concentration, presence of other molecules. However, the most common response is to temperature. Some thermo-responsive polymers demonstrate lower critical solution temperature (LCST). When the polymer chains and the solvent molecules are below LCST, they exhibit a homogenous mixed phase, whereas the separation of phase occurs above LCST. Some of the well-known thermo-responsive polymers are poly[N-[2-(diethyl amino)ethyl acryl amide]] (PDEAEAM), poly(N,Ndiethylaminoethyl methacrylate) (PDEAEMA), poly(2-(Nmorpholine) ethyl methacrylate) (PMEMA), poly(N,Ndimethylaminoethyl methacrylate) (PDMAEMA), poly(oligo(ethylene glycol)methacrylate) and poly(N,N-diethylacrylamide) (PDEAAM) [136]. The applications of thermo-responsive polymers include drug and gene delivery systems, tissue engineering scaffold structures, biosensing smart coatings, etc. The thermo-responsive polymer is classified into hydrogels, interpenetrating networks, micelles, polymersomes, particles, and films based on their structure, architecture and morphological state.

Zhou et al. [137] developed a heat-sensitive polymer polyvinyl alcohol (PVOH) using a material extrusion additive manufacturing method. The fabrication was carried out using a combination of compounding, extrusion, and additive manufacturing. They used PVOH granules and extruded them with minimum processing temperature. Yuan et al. [138] proposed a fabrication methodology to prepare CNT composite powders and fabricate them using SLS so that the composites have electrical and thermally conductive properties. Du et al. [139] demonstrated the fabrication of carbon black/B12Te3 based alloy with PLA composites using AM. Their thermoelectric properties were investigated from 300 to 360 K and found to be satisfactory. Shimizu et al. fabricated a cell sheet-polymer film complex with good strength. These composites can be removed after transplantation by lowering the temperature. However, these composites with ECM proteins do not have high stability when they are stored for the long-term. To overcome this disadvantage, they used oxygen plasma treatment on thermo-responsive poly-N-isopropylacrylamide [140]. Constantine et al. demonstrated the fabrication of copper/diamond composites using additive manufacturing. It was observed that the fabricated composites have good thermal properties [141].

### 3.4. Thermoplastic Elastomers Material Composites

Elastomers are polymers, which have weak intermolecular forces. They undergo a reversible response to high strain when subjected to force [142]. Thermoplastic elastomers (TPEs) enable the production of rubber-like articles using AM processes. They have rubber-like physical properties such as softness, flexibility, and resilience. Thermoplastic elastomers are classified into six types namely, multiblock copolymers, ionomers, hard polymer elastomer combinations, styrenic thermoplastic elastomers, graft copolymers, and core-shell morphologies. Styrenic thermoplastic elastomers are based on simple molecules such as A–B–A block co-polymer. Here ‘A’ refers to polystyrene and ‘B’ refers to the elastomer. Multiblock copolymers are elastomers having structures in the form of A–B–A–B–A–B–A–B or (A–B)_n_. In this ‘A’ is hard crystalline thermoplastics and ‘B’ is a soft amorphous elastomer. In most of these types of TPEs, ‘A’ segments are thermoplastic polyesters, thermoplastic polyurethanes, or thermoplastic polyamides and the ‘B’ segments are either polyethers or polyesters. Hard polymer elastomer combinations are fine dispersion of thermoplastic polymers and elastomers. Graft polymers are represented by the following structure.

(1) $$\begin{array}{c} \mathrm{B-B-B-B-B-B . . . B}\\ |\\ {\left(A\right)}_{n} \end{array}$$

This represents a polymer in which each elastomeric ‘B‘chain consists of n random grafts of ‘A’ blocks. ‘B’ chains with less than two ‘A’ blocks will not be elastically effective, because they will not be able to form a continuous interlinked network [143]. Wang et al., [108] printed viscoelastic nanoparticle suspensions with elastomers that are tough using digital light synthesis process. In this work, functional silica nanoparticles were dispersed in a poly (dimethylsilixane) matrix. The built structures are found to be tougher than those formed from the plain polymers [108]. Thermoplastic polymers are widely used in applications in electronics and medicine. Thus the possibility of using these new materials accelerates the implementation of innovative products [144].

### 3.5. Additive Manufacturing of Multi-Material Structures

Apart from the capability of 3D printing techniques in manufacturing 3D objects with complex geometries; another important potential is its capability of using different materials in a single manufacturing platform to fabricate multi-material objects and composites. Due to the implementation of multi-material manufacturing, it is observed that these products have improved functionality and weight reduction [145]. Therefore, the overall performance of the manufactured components is improved. However, multi-material manufacturing has certain limitations. This includes dimensional accuracy and size, need for post-processing, inability to process multiple materials at the same conditions, etc. Multi-material additive manufacturing is carried out primarily by processes such as FDM, Polyjet printing, etc. [146].

Recently, research has been carried out using multicomponent biolinks, which consists of two or more biomaterials in order to achieve better printability, bio functionality, and shape fidelity [147]. Chen et al. [109] developed a pneumatic extrusion additive manufacturing system with multiple nozzles for the fabrication of parts with a combination of soft and hard material. With this system, they demonstrated the fabrication of a sandwich pad using FDM. Bahr et al. [110] demonstrated multi-material processing using stereolithography. This is carried out by interconnecting the inkjet printing of silver nanoparticle inks. Bruyas et al. [111] developed a multi-functional compliant joint for unibody robotic mechanisms using multimaterial additive manufacturing. Pa et al. [148] developed an antenna in which an artificial magnetic conducting ground plane is integrated using a multi-material AM process. In this work, standard FDM is combined with conductive microdispensing printing in order to produce a mechanically robust antenna system with dielectric properties and intricate 3D conducting networks. Mirotznik et al. [149] also fabricated an antenna using FDM. Nassar et al. [150] reported an additive manufacturing method to manufacture smart sensing structures with bending capability. They are developed using printed strain sensors so that embedded electronic components can be manufactured. The electrical connectivity was found to be excellent. Taylor et al. [151] demonstrated a multi-material miniature diaphragm pump using the polyjet printing technique. Gheisari et al., fabricated 3D communication components and sensors using slurry based multi-material additive manufacturing and selective laser burnout [152]. Muguruza et al. [153] employed a hybrid printing machine, which combines digital light printing process with a drop-on demand inkjet printing system for manufacturing multi-material electronic devices. Singh et al. [154] analyzed multi-material manufacturing using three different thermoplastics namely ABS, PLA, and high impact polystyrene (HIPS) using FDM. From the results, it was observed that these materials are suitable for applications that require load-bearing capability.

## 4. Influence of Processing Parameters on Printed Product Quality

Material characteristics and process parameters have a significant impact on product quality and performance. Most of the investigators have suggested optimization of a variety of process parameters including the diameter of the nozzle, temperature of the envelope, layer thickness, temperature of extrusion, filler particle size, extrusion velocity, raster orientation of the part while building, raster angle, road width, filling velocity and raster gap to optimize the product’s quality, accuracy in dimension and good mechanical properties [155]. The process parameters considered for FDM include layer thickness, which refers to the height of each slice of the additively manufactured part; raster angle, which refers to the angles at which the nozzles deposits thermoplastics in a molten state; contours or shell perimeters, which refers to the outermost shells that form the outer skin and the interior hole of the part; raster width, which refers to the width of the extruder filament; raster gap, which refers to the gap between two adjacent extruded filaments; the speed of deposition, which is the speed of movement of the nozzle; fill density, which is the amount of materials within the part. Moreover, platform temperature, chamber temperature, and build orientation are also important. These issues have been discuss in detail in recent publications for specific polymer materials [156,157,158].

One of the most important critical necessities of additively manufactured parts especially is good layer adhesive property. For instance, the parts used in electronic industries are exposed to harsh environmental conditions such as humidity extremes, vibration, temperature, and shock. In such cases, the adhesion of each layer with the other is very important. However, only a few methods are available for measuring the adhesion of additively manufactured components. The adhesion property can be assessed with coating adhesion tests such as scotch tape testing and cross-hatch scratch testing. However, they provide only adhesion indications that are qualitative. A single lap test is used for the measurement of the bond strength of an adhesive that connects two layers that overlap each other [159]. Garcia et al. [160] explored the adhesively bonded joints using a single lap testing. The parts which were manufactured using FDM were tested. It was observed that parts manufactured using FDM resulted in average peak loads and shear strength than the parts manufactured using conventional adhesive methods. Neff et al. [161] analyzed the adhesive properties of electronic parts manufactured using additive manufacturing. Scratch adhesion test and single lap shear testing are generally carried out in order to determine the shear strength to analyze the adhesive property. Angelo et al. [162] studied the property of adhesiveness of composites that are made of poly (3, 4-ethylenedioxythiophene), poly (styrene sulfonate), and carbon nanotubes. The peeling of an adhesive tape from the surface of the samples that were printed was evaluated using an optical microscope. It was observed that the conductive ink was porous. However, they remained as a film on the surface. Freund et al. [163] proposed a systematic approach to identify and quantify adhesive property that influences interface strength. Harris et al. [164] investigated the adhesion in multi-material additive manufactured products made of ABS or ASA and TPU. Lap shear adhesion tests were conducted, and it was found that additively manufactured parts were equivalent to commercial adhesives and can be used as an alternative to industry-relevant handmade fabrication. Harris et al. [165] recently reviewed the process specific factors on the strength of printed parts in fused filament fabrication

Asif et al. [166] investigated the interfacial adhesion effects of composited manufactured using photopolymer additive manufacturing technique using a prediction model and SEM images. Malengiar et al. [167] investigated the methods to quantify adhesion in the textile industry where PLA parts were 3D printed on textile substrate. The three methods were the perpendicular tensile test, shear test, and peel test. These methods were identified to help in standardizing 3D printing on textiles. Similarly, Mpofu et al. [168] also investigated the fabric properties that affect the adhesion of PLA on woven fabrics such as cotton, polyester, and acrylic. It was observed that fabric areal density, weft count, fabric thickness, warp count, and fabric handle have a good correlation with PLA adhesion, whereas warp ends, and weft picks have a poor correlation with the PLA adhesion with woven fabrics. Elkins et al. [169] have also investigated the adhesive bonding between metal and polymer of composite made of Grade 5 Ti-6AL-4v and carbon fiber reinforced PPS. Overall, interlayer adhesion has a strong influence in controlling the final part properties.

## 5. Application of 3D Printed Polymer Products and Composites

Additively manufactured polymer composites are widely used in various industries including construction, biomedical, electronics, aerospace, and textile industries. They are also used to manufacture flexible parts such as wearable electronics and textile materials. Research undertaken in various fields of applications are discussed below.

### 5.1. Biomedical Applications

AM using polymeric materials are used widely in biomedical applications, where customized tools, therapies, medicines, and organ replacements are required. Polymer-based materials that are used in biomedical applications are classified into hard and soft polymers. They are further classified into two types namely biodegradable and non-biodegradable polymers. Biodegradable polymers are used in applications, where the tissues grow and the biomedical parts inside the body are not required once the task is complete. They are degraded in the human body such as hydrolysis of the ester linkages in case of polyesters. However, non-biodegradable polymers are used as structural implants. Some of the hard-synthetic biodegradable polymers that are used in biomedical applications include polylactic acid (PLA), polycaprolactone (PCL), polyglycolic acid (PGA), and polydioxanone (PDO). Some of the non–biodegradable hard polymers used include the polymers of polyaryletherketones (PAEKs). Some of the biomedical applications of hard polymers are implants, dental implants, surgical models, and prosthetics. Some of the soft polymers used in biomedical applications are polyethylene glycol (PEG), acrylate-based hydrogels, elastomers, and hydrogels with unique thermal properties such as poloxamer pluronic F127. Soft polymer biomedical applications include tissue engineering and bioprinting, drug delivery, tissue phantoms, and soft surgical models [92]. Ho et al., [170] developed additively manufactured scaffolds made of polycaprolactone and CNTs for cardiac tissue engineering. Ramirez et al., [171] developed an additive manufacturing technique for manufacturing composite materials using FFF/FDM fabrication in order to improve mechanical properties. Jockusch et al. [172] have discussed the applications of additive manufacturing in dental applications such as study models, maxillo-facial, prostheses, and orthodontic appliances.

### 5.2. Applications in the Electronics industry

Additive manufacturing has promising applications in embedded electronics. There are two approaches that are used. In the first approach, the discrete components are prefabricated and are transferred using laser directly into a substrate; whereas, in the second approach the functional components like interconnects, actives and passives are laser printed on the substrate [173]. Goh et al. [174] have discussed 3D printing of carbon nanotube-based materials in order to manufacture functional electronic parts. Tao et al. [175] developed supercapacitors that are flexible with polypyrrole-MnO_2_-carbon fiber hybrid structures using AM. Such composite active materials have huge potential in energy management. Wang et al. [176] developed a thin flexible pressure sensor with carbon black and silicone rubber nanocomposite. Similarly, Tadakaluru et al. [177] reported high-strain sensors that are flexible and stretchable. These consist of CNTs that are randomly distributed or graphite flakes on natural rubber substrate. Mirzaee et al. [178] investigated the feasibility of conductive ABS for the manufacturing flexible 3D antennas using additive manufacturing.

### 5.3. Aerospace Applications

AM such as laser-based 3D printing processes is used widely in aerospace and defense industries due to their ability to produce complex geometries and reduce development costs. The most popular polymer-based AM processes used in aerospace applications include SLA, FDM, and Jet type processes such as polyjet, etc. It is observed from the research undertaken that parts made using SLA are sturdier than the ones made using FDM and therefore SLA based products are best suited for casting purposes in the aerospace industry [179]. The aerospace industries require parts made of materials with lower weight and high strength, which is possible using AM and can be manufactured on demand. NASA has designed a Rover called Desert RATS that contains about 70 additively manufactured parts. These parts include flame-retardant vents and housings, large pod doors, front bumpers, camera mounts, complex electronics, etc. They have used parts made of ABS, PC, and PCABS which were fabricated using the FDM process [180]. Goh et al. [181] have explored the applications of AM for unmanned vehicles. AM’s potential in unmanned vehicles include printing using multiple materials and smart materials, printing on-site on-demand, multifunctional structures, etc. Polymer nanocomposites (PNCs) have obtained much attention in aerospace applications. PNCs have nanofillers that are of different shapes such as platelets, fibers, etc., in polymer matrix [182]. Polymer nanocomposites provide increased modulus, thermal performance, resistance to molecule penetration, atomic oxygen resistance, and improved ablative performance when compared with conventional composites [183]. The commonly used nanocomposites for aerospace applications are carbon nanotube, graphene, graphene oxide, and clay [184]. However, these nanocomposites do not meet the requirements of strength necessary for aerospace structures. Therefore, hybrid composites that combine nanocomposites and conventional composites are developed and are known as multiscale composites [185]. William et al. [186] have developed self-healing carbon fiber reinforced composites in order to address the effects of damage in composite materials in the aerospace applications.

### 5.4. Applications in Textile Industries

Additive manufacturing is used in textile industries due to its potential to improve complexity and functionality. They also enable designing and developing novel solutions with better performance textile applications [187]. While using AM for manufacturing textiles, the primary aim must be the ability to reproduce the textile properties that are important such as softness, strength, flexibility, and porosity [188]. AM of textiles provides customized clothing. They are also capable of carrying out designated tasks in producing components that transform from solids to textiles such as optimized footwear and give textiles added functionality through design [189]. Korger et al. [190] investigated 3D printing on textile substrates. As adhesion and good stability are very important in 3D printing on textile substrates, separation force and abrasion resistance tests were conducted in order to demonstrate that sufficient adhesion is achieved. Melnikova et al. [191] investigated the manufacturing of textile-based structures using FDM using various polymers namely ABS and PLA. Pei et al. [192] investigated the adhesion of various polymers such as ABS, PLA, and nylon on different types of fabrics and observed PLA has the best adhesion properties. Johnson et al. [193] explored additive manufacturing of textiles for high-performance stab resistant characteristics using articulated laser sintered samples. Leist et al. [194] investigated shape memory polymers in AM of textiles. In this work, a combination of PLA with nylon fabric was used to create smart textiles, which can be thermo-mechanically trained to form various shapes and return to their original shapes when they are exposed to high temperature. Maiti et al. [195] developed non-metallic textiles that are flexible and electrically conductive with polymers such as aniline, pyrrole, thiophene, and their derivatives as shown in Table 6.

## 6. 4D Printing

4D printing is a unique extension of 3D printing, which enables changes in the shapes and the properties of the printed articles on a temporal (time) basis. 4D printing, first highlighted in 2013, and it enables the development of structures that are dynamic and capable of undergoing shape transformations and have a great capacity for fabricating complex items. 4D printing has demonstrated its potential to fabricate smart components for self-repairing, self-assembly, and self-adapting [199]. This new process refers to the integration of 3D printing with smart materials to develop printed components that can change to multiple configurations in response to the environmental stimulus. The environmental stimuli may include temperature, chemical agents, radiation, mechanical stress, pH, and electric and magnetic fields [200]. The materials that are commonly used for 4D printing are hydrogels and shape memory polymers [201]. 4D printing has many hurdles that it must overcome to achieve its full potential in the manufacturing technology. Some of the major hurdles include material’s mechanical strength reduction, longer time of response to stimuli which results in a slow rate of shape changes [202].

**Figure 5:**
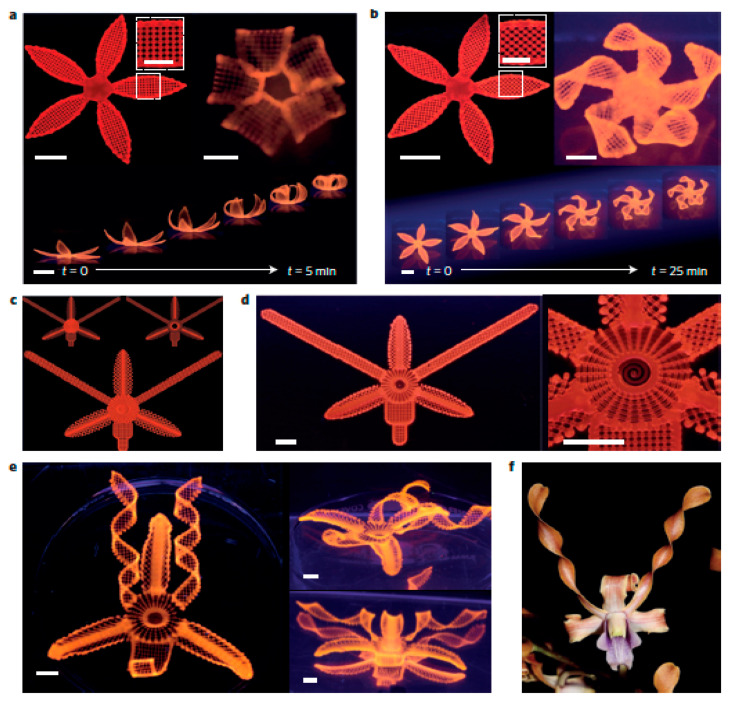
Complex flower morphologies generated by biomimetic 4D printing. (**a**,**b**) Simple flowers composed of 90°/0° (**a**) and 45°/45° (**b**) bilayers oriented with respect to the long axis of each petal, with time-lapse sequences of the flowers during the swelling process (bottom panel) (scale bars, 5 mm, inset = 2.5 mm). (**c**–**f**) Print path (**c**), printed structure (**d**) and resulting swollen structure (**e**) of a flower demonstrating a range of morphologies inspired by a native orchid, the Dendrobium helix (**f**). Based on the print path, this orchid architecture exhibits four different configurations: bending, twisting and ruing corolla surrounding the central funnel-like domain (scale bars, 5 mm). Reprinted by permission from [208] published by Springer Nature.

There are four major approaches for 4D printing namely (i) self-assembly of elements, (ii) bi-stability, (iii) deformation mismatch, and (iv) shape memory effect [203]. Self-assembly of elements enables a 3D printed component to automatically assemble itself when certain conditions are applied. This method is used especially in biomedical applications. For example, it enables the delivery of active components inside the human body through a small hole and thereafter assembles them inside the body during the surgery [204]. In the bi-stability approach, structures with zero degrees of freedom and also have two or more stable positions under some conditions are used. Such structures have the capability to switch from one stable position to another while it is properly induced for slight deformations [205]. Deformation mismatch is another approach for 4D printing. In this method, some physical properties such as swelling ratio and coefficient of thermal expansion are used to induce the deformation mismatch. Some materials are capable of recovering their original shape after being deformed when the right stimulus is applied, which is known as shape memory effect (SME) [206]. In some materials, the magnitude of the shape change is proportional to the stimulus that is applied. These are called shape change effect (SCE) [207]. Such materials are used in AM to obtain shape memory effect, thus the added dimension to 4D printing. Gladman et al. (211) demonstrated that 4D printing path ways can be employed in a predictable way to fabricate complex composite hydrogel architectures (Figure 5), which was inspired by botanical systems. The final Complex flowermorphologies generated were encoded into the 3D printed composite hydrogels using localized, anisotropic swelling behaviour- controlled by the alignment of cellulose fibrils.

Many researchers have studied 4D printing technology for various applications. Ly et al. developed smart and wearable textile products using the 4D printing process exploiting shape memory polymers and carbon nano-composites [209]. In the electronics field, electronics devices used in applications such as human/machine interfaces, wearable electronics, and soft robotics are developed using 4D printing [210]. In order to develop electronic devices using 4D printing, electrically functional materials are used for the printing process [88,211]. In the bio-medical field, 4D printing is used to develop tissues and organs using cells, biological molecules, and biomaterials [212]. Moreover, it also explores biomedical devices that can be used to treat inaccessible areas of the human body [213]. Kashyap et al. [214] developed a radio-opaque, porous, and custom-shaped shape memory polyurethane that can be applied in endovascular embolization. Morrison et al. developed stents, which were implanted in air passages to support breathing in children [215]. Further 4D printing is also applied in origami to mimic flowers, buds leaves, etc. Manen et al. demonstrated the progression of folding of leaves of Mimosa pudica, etc. [216].

In the future, 4D printing has to be further developed for application in various fields. Moreover, 3D printing hardware, smart materials, novel designs, and modeling tools have to be developed further along with machine learning to obtain/optimize high speed, high resolution, and multifunctional material printing techniques [217].

## 7. AM of Novel, Digita, l and Smart Materials

Novel materials are a collection of advanced materials that can be 3D/4D printed for specific applications. Digital material is defined as an advanced composite material with two or three photopolymers with specific microstructures and ratios. These materials are used to fabricate functional prototypes with characteristics that can be customized. These characteristics include superficial hardness, textures, and colors. Smart materials are the materials that can transform their geometry when they are subjected to external stimuli [218]. The material complexity allowed by AM has made smart materials (SMs) processing easier than usual [219]. Recent research has focused on the development and manufacturing of these digital and smart materials for various applications. Caputo et al. [220] demonstrated the manufacturing of functional parts that are net shaped. They are made of pre-alloyed shape-memory Ni-Mn-Ga powders with magnetic properties. Powder bed binding jetting was used in this research. Bakarich et al. [221] investigated the performance of thermally actuated hydrogels, which was fabricated using AM techniques and it was observed to have good mechanical properties. Smart materials like shape memory alloys are thermo-mechanically programmed in order to obtain 3D configurations with complex geometries such as bents, coils, twists, folds, etc. The flat shape that is present initially can be recovered again by exposing the material at high temperatures [222]. Raviv et al. proposed active self-evolving structures [223]. These structures transform into a shape that is predetermined and changes its properties and functions after fabrication. The developed smart material can deform under the environmental stimulus. Woodward et al. [224] reported the fabrication of dense piezoelectric ceramic components using additive manufacturing methods. In this work, 0.65Pb(Mg1/3Nb2/3)O3–0.35PbTiO3 piezoelectric material was built and was demonstrated to generate ultrasound in the MHz range. Yu et al. [225] demonstrated the fabrication of shape memory polymers that are functionally graded using 3D printing. The manufactured components react to temperature and return to the specified configuration in a controlled shape-changing sequence. It was observed that 3D printing enables easy implementation, better freedom of design, and high printing resolution.

## 8. Conclusions and Future Prospects

This article reviews recent advances and achievements in the field of different AM processes, for polymers and their composites with functional materials, elastomers, shape memory polymers, and thermo-responsive materials. The current capabilities of additive processes and optimization tools are summarized and it has been reflected that AM of polymers, composites, and functional materials appears to be promising in converting 3D printing from a prototyping method to a robust manufacturing process. Novel tools and techniques in AM have been developed that enable innovative ways to print plastic/composite structure faster, bigger and weirder and developing innovative ways to create stronger materials, smarter ways; to 3D-print intricate resins, print polymer materials endowing with conflicting properties, and even mixing multiple materials in the same product. Moreover, to improve the intrinsic strength of printed metals, sometimes by controlling the microstructures of the materials. Moreover, the different parameters that control the mechanical characters such as modulus and flexural strength and adhesion properties are also reviewed. Applications of AM in the fields of construction, electronics, biomedical, aerospace, and textile industries have also been reported.

In 2020, AM has established itself as a highly versatile manufacturing methodology in which ‘complexity is free’ with flexibility; which has opened a world of new possibilities and ‘consequential technologies in the fourth industrial revolution’ and has demonstrated the potential to completely transform traditional manufacturing in future. These innovations are broadening the prospects and opening a new world of design, research, and manufacturing using AM technology that was once viewed as useful only for making small, low-quality prototypes. [225,226,227,228,229]. It is offering designers and engineers the opportunity to push boundaries to get performance out of polymer materials and composite materials that thought not been possible. AM strategy opening new flexibility and possibilities of fabricating bioinspired composites and electrodes with contradicting properties such as both high flexural strength and hierarchical porous structure. AM manufacturing offers unprecedented opportunities to design complex structures optimized for performance envelopes inaccessible under conventional manufacturing, allows industrial designers to think differently; to reimagine products and components in new ways. It can be predicted that the expansion and evolution of 3D printing technology itself will bring exciting new developments and manufacturing-ecosystems, colonize a wider range of environments, reshape global supply chains, dramatically reduce production losses, and even strengthening local networks [230,231]. In building/construction industries, computers and robots have been used to precisely automate the pouring and 3D printing of concrete and have engineered large structures such as 3D printed demonstration houses and functional bridges. AM has the potential to build houses not only low in cost and more efficient but also to reduce concrete’s carbon footprint. Aviation firms such as Boeing, Rolls Royce, and Pratt & Whitney are using AM to make metal parts for jet engines that can be cheaper than milling metal blocks, and the intricate components often weigh less than their conventionally made counterparts.

4D printing has created new opportunities in engineering smart objects in many areas including biomedical and medicine, aerospace, manufacturing, robotics, and the future possibilities of this technology are seemingly unbounded [232,233,234,235,236,237,238,239,240,241,242,243]. 4D printing shows potential in the fabrication of actuators and sensors for engineering applications to way into mainstream art and design for use within the field of smart textiles such as orthopedic casts, in wearable technologies and activewear. It has potential for modular fabrication of intelligent material-tissue interfaces for bioinspired and biomimetic devices [241]. This technology shows interesting applications within the field of retail and e-commerce. With the realization of 3D/4D printing AM has the potential to become more versatile, able to handle and combine an expanding variety of materials and complex shapes never thought possible before in a rather simple way-biomimetically. Sustainable and cost-effective solutions are crucial for the widespread adoption of 4D printing technology. The major limitations of this technology are the availability of the limited number of smart materials and suitable for printing- especially those which are biocompatible and reduced fabrication costs [238,239,240,241,242]. For biomedical applications it is necessary to develop new smart materials that is not only bio-compatible but also display multiple stimuli-responsiveness [208,243,244].

However, much potential of additive manufacturing methods still needs to be explored and investigated in the areas of manufacturing and design development. Moreover, the development of new functional materials and new processes to sustain AM as a robust manufacturing method has to be carried out. In the case of TPEs, novel materials can be prepared using blends of elastic polymer with dimensional stability, and ionomers such as carboxylates, sulfonates and phosphate can be included. In the case of stimuli-responsive polymers, developments are necessary with new materials that have the ability to meet the needs of increasing strength, flexibility, texture, and other qualities that continue for diverse applications. In the future, effective integration of additive manufacturing techniques with other manufacturing techniques may overcome the challenges in the selection of materials, control, functionality, etc. Furthermore, multi-process, multi-scale additive manufacturing will show many advances in the capacity of materials and structures and functionalities of the parts produced. The AM process is easy—and yet so powerful—and the possibilities are endless for product development and manufacturing, and it has potential in applications in all four main categories: concept models, functional prototypes, manufacturing tools and finished goods, that both hobbyist’s basement to home-based businesses to Fortune 500 companies can count on. While numerous challenges and opportunities exist before realizing this future vision; concerted, collaborative research and development efforts across additive and optimization communities will be crucial to sustain momentum and guide progress.

## Figures and Tables

**Table 1:** Polymer additive manufacturing process and global 3D-printer manufacturers.

Methodology of forMation of the Product	Polymer AM Process Technique	3D-Printer Manufacturer with Headquarter	Polymer Used
Matrix Extrusion	Fused deposition modeling, FDM	StrataSys, USA Ultimaker B.V., Netherlands Bigrep, Germany Makerbot, USA Markforged, UK Raise-3D, USA Tractus-3D, Netherlands Zortrax SA, Poland Roboze, Italy Intamysys, China	ABS, PEEK, PC, PC-ABS, PLA, Nylon 12/Carbon Fiber, HIPS, TPU, ASA, PEEK, PEI
Vat Polymerization	Stereolithography, SLA	3D-Systems, USA Formlabs, USA Carbon, USA Prodways, France Envisiontec, USA Asiga, Australia Photocentric, UK Nexa 3D, USA Origin, USA	liquid UV-curable photopolymers Somos^®^ stereolithography (SLA) materials Somos^®^ 9120 (off white), Somos^®^ BioClear (clear), Somos^®^ Element (clear), Somos^®^ EvoLVe 128 (white), Somos^®^ NeXt (white), Somos^®^ WaterClear Ultra 10122
Powder Bed Fusion	Selective laser sintering, SLS	Eos, GbbH, Germany 3D-Systems, USA Formlabs, USA Prodways, France Sinterik SA, Poland Farsoon Technologies, China	Polymer in powder form polycarbonate (PC) nylons (polyamide (PA)), acrylic styrene (PMMA/PS), polyamides(PA), polystyrenes(PS), thermoplastic elastomers(TPE), polyaryletherketones (PAEK).
	Multi jet Fusion	Hewlett-Packard, USA	Polyamide (PA12/Nylon 12), Elastic TPU
Material Jetting	Material Jetting	Objet, USA 3D-systems, USA Mimaki, Japan	Vero, Tango, Durus, Digital ABS

**Table 2:** Properties of commercial polymers used in additive manufacturing processes.

Properties	ABS	PLA	PC	Nylon 12	PA 11	PA 12	Protogen O-XT 18240	Water Clear Ultra 10122	Durus White	Vero	RGD 525
Tensile Modulus (MPa)	1627	2347	1944	1282	1600	1650	2960	2880	1200	3000	3500
Tensile Strength (MPa)	22	50	40	32	48	48	68	56	30	65	70
Density (kg/m^3^)	1050	1240	1200	950	990	930	1160	1130	1170	1190	1180

**Table 3:** Techniques and properties enhanced in polymer composites.

Technique	Materials	Enhancement in Properties	Reference
Large Format Additive Manufacturing (LFAM)	Acrylonitrile Styrene Acrylate (ASA)	Higher performance of the CF loaded composite compared to the raw ASA polymer (i.e., the 20 wt % CF composite shows a 350% increase in flexural Young’s modulus and a 500% increment in thermal conductivity compared with neat ASA).	[102]
FDM	Polypropylene	The results showed the potential of the FDM to compete with conventional techniques, especially for the production of small series of parts/components; also, it was showed that this technique allows the production of parts with adequate mechanical performance and, therefore, does not need to be restricted to the production of mockups and prototypes	[104]
Digital Light Synthesis	Functionalized silica nanoparticles suspended in a poly(dimethylsiloxane) matrix	The border of printability at standard temperature and pressure (STP) is established by resin with a silica nanoparticle mass fraction of 0.15.	[108]
Multi-nozzle additive manufacturing system	Sandwich pad with soft and hard material structure	A finer printing performance than a traditional FDM machine is achieved.	[109]
Multimaterial Stereolithography	3D printed multi-chip module with an on-package enhanced dielectric lens for mm-wave applications	The ability to 3-D print multiple materials of different dielectric constants at optical resolutions enables the formations of entirely new structures to be integrated into system-on-package solutions for mm-wave applications.	[110]
Multimaterial additive manufacturing	A new multifunctional compliant joint for unibody robotic mechanisms.	It offers interesting performances while being compact and MR-compatible	[111]

**Table 4:** Techniques used and properties enhancement in particle reinforced polymer composites.

Technique	Materials	Enhancement in Properties	Reference
Stereolithography	Graphene oxide/polymer composites	The method presented in this paper proved to be successful for producing designed 3D structures but further optimization is needed for practical applications due to the high shrinking and brittleness of the pyrolyzed 3D constructs. By pyrolyzing the polymer component only partly, electrical conductivities in the range of semiconductors were achieved.	[114]
Reversible thermal- and shear-induced dissociation of a supramolecular polymer network	Linear and three-arm star supramolecular polymers with attached hydrogen bonds and their nanocomposites	The supramolecular PIB polymers show a rubber-like behavior and can form self-supported 3D printed objects at room temperature and below, reaching polymer strand diameters down to 200–300 μm.	[115]
SLM	Auxetic foams composed of highly porous thermoplastic polyurethane (TPU)	Highly recoverable, undergoing repeated compressions, and they retained the auxetic properties over a wide range of applied deformations.	[117]

**Table 5:** Techniques used and properties enhanced in fiber reinforced polymer composites.

Technique	Materials	Enhancement in properties	Reference
Dynamic capillary-driven AM approach	Carbon fiber composites	High fiber volume fraction (58.6%) and degree of curing (95%) with high mechanical strength (810 MPa) and modulus (108 GPa).	[118]
Direct write AM	Short fiber reinforced thermoset composites	High compression strength (673 MPa), flexural strength (401 MPa), flexural stiffness (53 GPa), and fiber volume ratio (46%)	[119]
FDM	Continuous carbon, Kevlar, and glass fiber reinforced composites	Maximum efficiency in tensile strength was observed in glass specimen as fiber content approached 22.5%, with higher fiber contents (up to 33%), yielding only minor increases in strength.	[121]

**Table 6:** Applications of electrically conductive polymers.

Polymer	Applications	Reference
Polyaniline	Ammonia sensor, electronic devices	[196]
Polypyrrole	Heating pads, EMI shielding, sensors, actuators, antistatic materials	[197]
Polythiophene	Microwave attenuation, static charge dissipation, and EMI shielding	[198]

## Data Availability

No new data were created or analyzed in this study. Data sharing is not applicable to this article.

## Acronyms

AA	Ascorbic acid
ABS	Acrylonitrile butadiene styrene
AM	Additive Manufacturing
ASA	Acrylonitrile styrene acrylate
ASTM	American Society for Testing and Materials
BJ	Binder Jetting
CAD	Computer Aided Design
CFF	Composite filament fabrication
CNC	Computer Numerical Control
CNTs	Carbon Nano Tubes
DED	Directed Energy Deposition
DMD	Direct Metal Deposition
DLP	Direct Light Processing
dECM	Decellularized extracellular matrix
EBM	Electron Beam Melting
EBAM	Electron Beam Additive Manufacturing
EPCs	Endothelial progenitor cells
FRC	Fiber reinforced composites
FEAM	Fiber Encapsulation Additive Manufacturing
FDM	Fused Deposition Modeling
FFF	Fused Filament Fabrication
HIPS	High-impact polystyrene
LAPS	Light-addressable potentiometric sensors
LDM	Liquid deposition modeling
LENS	Laser Engineered Net Shaping
LOM	Laminated object manufacturing
LIG	Laser-induced graphene
LCST	Low critical solution temperature
MEMS	Microelectromechanical systems
MJF	Multi Jet Fusion
MJ	Material Jetting
PBF	Powder Bed Fusion
PEKK	Polyetherketoneketone
PLA	Polylactic acid
PA	Polyamide
PEEK	Polyether ether Ketone
PEI	Polyetherimide
PSL	Plastic Sheet Lamination
PEGDMA	Poly(ethylene glycol) dimethacrylate
PLLA	Poly L-lactic acid
PS	Polystyrenes
PMMA	Polymethylmethacrylate
PMC	Polymer matrix composites
PDMS	Polydimethylsilixane
PDEAEAM	Poly[N-[2-(diethyl amino)ethyl acryl amide]]
PCL	Polycaprolactone
PGA	Polyglycolic acid
PEG	Polyethylene glycol
PNC	Polymer nanocomposite
SLS	Selective Laser Sintering
SLM	Selective Laser Melting
SLA	Stereolithography
SHS	Selective Heat Sintering
TPU	Thermoplastic polyurethane
TPE	Thermo plastic elastomers
UV	Ultraviolet
UAM	Ultrasonic additive manufacturing
3D	Three-dimensional
3DP	Three-dimensional Printing
4D	Four-dimensional
